# Single-mode dispersive waves and soliton microcomb dynamics

**DOI:** 10.1038/ncomms14869

**Published:** 2017-03-23

**Authors:** Xu Yi, Qi-Fan Yang, Xueyue Zhang, Ki Youl Yang, Xinbai Li, Kerry Vahala

**Affiliations:** 1 T.J. Watson Laboratory of Applied Physics, California Institute of Technology, Pasadena, California 91125, USA; 2 Department of Microelectronics and Nanoelectronics, Tsinghua University, Beijing 100084, China; 3 State Key Laboratory of Advanced Optical Communication Systems and Networks, School of Electronics Engineering and Computer Science, Peking University, Beijing 100871, China

## Abstract

Dissipative Kerr solitons are self-sustaining optical wavepackets in resonators. They use the Kerr nonlinearity to both compensate dispersion and offset optical loss. Besides providing insights into nonlinear resonator physics, they can be applied in frequency metrology, precision clocks, and spectroscopy. Like other optical solitons, the dissipative Kerr soliton can radiate power as a dispersive wave through a process that is the optical analogue of Cherenkov radiation. Dispersive waves typically consist of an ensemble of optical modes. Here, a limiting case is studied in which the dispersive wave is concentrated into a single cavity mode. In this limit, its interaction with the soliton induces hysteresis behaviour in the soliton's spectral and temporal properties. Also, an operating point of enhanced repetition-rate stability occurs through balance of dispersive-wave recoil and Raman-induced soliton-self-frequency shift. The single-mode dispersive wave can therefore provide quiet states of soliton comb operation useful in many applications.

## Abstract (web summary)

Dissipative Kerr solitons in microresonators have recently been shown to generate frequency combs via Cherenkov radiation. Here, Yi *et al*. demonstrate hysteresis behaviour and a single-mode dispersive wave that can improve the stability of microcombs.

A new dissipative soliton^1^ has recently been observed in optical resonators. These dissipative Kerr solitons (DKS) have been demonstrated in fibre resonators^2^ and in various microcavity systems^3^,^4^,^5^,^6^,^7^. In microcomb research^8^,^9^ soliton formation produces phase-locked spectra with reproducible envelopes, as required in frequency comb applications^10^,^11^,^12^,^13^,^14^. Moreover, their unusual properties and interactions create a rich landscape for research in nonlinear optical phenomena^5^,^15^,^16^,^17^,^18^,^19^,^20^,^21^,^22^,^23^,^24^. Two such phenomena, the Raman-induced soliton-self-frequency-shift (SSFS) and dispersive-wave generation, are important to this work.

The Raman SSFS causes a spectral red shift of the soliton. In optical fibre systems, this shift continuously increases with propagation distance^25^,^26^, however, in microresonators the shift is fixed and depends upon soliton power^17^,^18^,^27^,^28^. Dispersive waves also occur in optical fibre systems^29^ where they are an important process in continuum generation^30^ and have been used to study general nonlinear phenomena^31^. They are formed when a soliton radiates into a spectral region of normal dispersion and can be understood as the optical analog of Cherenkov radiation^32^. In microcavities, dispersive waves provide a powerful way to spectrally broaden a soliton within a microresonator as a precursor to self referencing^12^,^33^. Their formation also induces soliton recoil^32^ which, similar to SSFS, causes a frequency shift in the spectral centre of the soliton^5^,^17^. Dispersive waves normally consist of an ensemble of modes that are phase matched to a soliton. This phase matching can be assisted by avoided mode crossings in microcavities^19^,^34^. Avoided mode crossings can also produce zero group velocity effects^35^,^36^, enable microcombs to form in regions of normal dispersion^37^, and provide a way to induce dark solitons^16^.

In this work, an avoided-mode crossing is used to excite a dispersive wave consisting of a single cavity mode. The coupling of this single-mode dispersive wave to the soliton is strongly influenced by the total soliton frequency shift produced by the combined Raman-induced SSFS and the dispersive-wave recoil. The combination is shown to induce hysteresis behaviour in soliton properties. Included in this behaviour, there is an operating point of improved pulse-rate stability (a quiet point) where the coupling of repetition rate and cavity-pump detuning is greatly reduced. Pulse-rate stability is centrally important in many frequency comb applications^10^,^13^,^38^. Coupling of pulse rate and cavity-pump detuning through avoided-mode-crossing recoil effects has been observed in crystalline resonators^39^. Also, the fundamental contributions to phase noise in the pulse train have been considered theoretically^40^. However, technical noise mechanisms are also present. For example, DKS generation using on-chip silica resonators exhibits phase noise that tracks in spectral profile the phase noise of the optical pump^4^. The quiet operation point is shown to reduce technical noise contributions to the soliton pulse repetition rate. Both this regime of operation and the hysteresis behaviour are measured and modelled theoretically.

## Results

### Mode family dispersion

A silica whispering-gallery resonator^41^ is used for soliton generation. The devices feature a free-spectral-range (FSR) of ∼22 GHz (3 mm diameter resonator) and have intrinsic *Q*-factors around 250 million. Specific details on soliton formation in these resonators are given elsewhere^4^,^42^. The resonators support multiple, transverse mode families. It is essential that the soliton-forming mode family feature dispersion that is primarily second-order and anomalous^43^. To characterize the frequency spectrum of the resonator, mode frequencies were measured from 190.95 THz (1,570 nm) to 195.94 THz (1,530 nm) using an external-cavity diode laser calibrated by a fibre Mach–Zehnder interferometer^4^. This provides a set of mode frequencies {*ω*_*μ*,*s*_} for each spatial mode family ‘*s*' with *μ* as the mode index.

**Figure 1:**
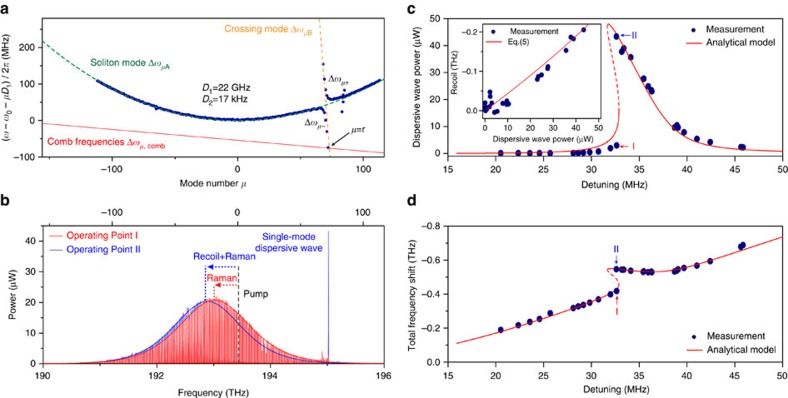
Soliton hysteretic behaviour induced by mode interaction. (**a**) Measured relative mode frequencies are shown as blue points^4^. The green and yellow dashed lines represent the fitted relative mode frequencies (Δ*ω*_*μ*A_ and Δ*ω*_*μ*B_) of the unperturbed soliton-forming mode family A and crossing mode family B, respectively. Relative mode frequencies for upper and lower branch hybrid-modes are Δ*ω*_*μ*+_ and Δ*ω*_*μ*−_. The red line illustrates the frequencies of a hypothetical soliton frequency comb. A non-zero slope on this line arises from the repetition rate change relative to the FSR at mode *μ*=0. (**b**) Measured optical spectra at soliton operating points I and II, corresponding to closely matched cavity-pump detuning frequencies, *δω*. A strong single-mode dispersive wave at *μ*=72 is observed for operating point II and causes a soliton recoil frequency shift. This frequency shift adds to the shift resulting from the Raman-induced SSFS. (**c**,**d**) Dispersive-wave power and soliton spectral centre frequency shift versus cavity-pump detuning. Operating points I and II of **b** are indicated. Inset in **c**: Measured (blue dots) and theoretical (red line) recoil frequency versus the dispersive wave power.

The mode family frequency data are presented in Fig. 1a by plotting the relative-mode-frequency, Δ*ω*_*μ*,*s*_≡*ω*_*μ*,*s*_−*ω*_0_−*μD*_1_ versus mode index *μ* where *ω*_0_ and *D*_1_ are specific to the soliton-forming mode family. *ω*_0_ is the frequency of the mode (set to have index *μ*=0) that is optically pumped to produce the soliton, and *D*_1_ is the FSR of the soliton-forming mode family at *μ*=0 (note: *μ* is a relative and not an absolute mode index). By plotting the data in this way the second- and higher-order dispersion of the soliton-forming mode family become manifest. To illustrate, the relative-mode-frequency of the soliton-mode family is fit with a green, dashed parabolic curve of positive curvature in Fig. 1a showing that it features anomalous second-order dispersion over a wide range of mode numbers.

A second mode family also appears in Fig. 1a and causes an avoided-mode-crossing near *μ*=72. Hybridization of this ‘crossing-mode' family with the soliton-mode family occurs near the avoided crossing^19^,^44^. The relative-mode-frequencies of the unperturbed soliton-forming mode family and crossing-mode family are denoted as Δ*ω*_*μ*A_ and Δ*ω*_*μ*B_. Over the range of mode indices measured Δ*ω*_*μ*A_=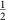
*D*_2_*μ*^2^ where *D*_2_ is the second-order dispersion at *μ*=0. The lower (upper) branch of the hybrid mode family is denoted by Δ*ω*_*μ*−_ (Δ*ω*_*μ*+_). The spatial modes associated with the soliton and crossing mode families are identified in the Supplementary Note 3. Avoided mode crossing behaviour has been intensively studied in the context of DKS formation and can interfere with soliton generation by creation of distortions in the dispersion spectrum^43^,^45^,^46^. In the present system, the avoided mode-crossing induces only minimal distortion in the otherwise parabolic shape of the soliton-forming mode family. Soliton spectra produced on this mode family by pumping at *μ*=0 are shown in Fig. 1b along with theoretical sech^2^ spectral envelopes predicted for DKSs. As an aside, the horizontal scales in Fig. 1a,b are identical and the location of the *μ*=0 pumping mode is indicated by a vertical dashed line in Fig. 1b.

### Single-mode dispersive-wave formation

Also shown in Fig. 1a are the comb frequencies associated with a hypothetical soliton spectrum plotted in the relative frequency frame. This comb line is given by,


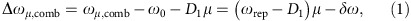


where *ω*_*μ*,comb_=*μω*_rep_+*ω*_p_ is the frequency of *μ*th comb line, *ω*_rep_ is the soliton repetition frequency, *ω*_p_ is the pump frequency, and *δω*≡*ω*_0_−*ω*_p_ is the cavity-pump detuning frequency. It is necessary to distinguish between relative frequencies for the soliton comb and the resonator modes because the frequency components of the soliton comb are strongly red-detuned relative to the cold-cavity mode frequencies by the Kerr nonlinearity. Indeed, dispersive waves typically form when a set of modes break this rule and become resonant with a set of comb lines. A limiting case of this condition is shown in Fig. 1a, where the occurrence of an isolated resonance between a hybrid mode with relative frequency Δ*ω*_r−_ and a comb line at Δ*ω*_r,comb_ is illustrated. The equation of motion for the hybrid mode field amplitude *h*_r−_ is shown in the Methods to have the following form,


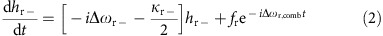


where *κ*_r−_ is its loss rate and *f*_r_ is an effective pumping term associated with the soliton comb line. The pumping term is given by *f*_r_=*i*Γ(Δ*ω*_rA_−Δ*ω*_r,comb_)*a*_r_, where Γ is the fraction of the family A mode in the hybrid mode, and *a*_r_ is the field amplitude of the unperturbed soliton hyperbolic solution at *μ*=r. Also, the Kerr-effect shift of *h*_r−_ is of order 10 kHz and is therefore negligible in comparison to *κ*_r−_.

Because the damping rate *κ*_r−_ is low (that is, the mode has a high optical *Q*-factor) slight shifts in the slope of the comb frequency line (equivalently, shifts of Δ*ω*_r,comb_ relative to Δ*ω*_r−_) will cause large changes in the power coupled to the hybrid mode. These changes are observable in Fig. 1b where a strong spectral line appears in the case of the blue soliton spectrum. Note that scattering from the soliton into the spectral line is strong enough so that the power in the line is greater than the comb line power near the spectral centre of the soliton, itself. The strong spectral line can be understood as a single-mode dispersive wave and it induces a recoil in the spectral centre of the soliton. This recoil contribution is indicated for the blue soliton spectrum in the figure. In the case of the red soliton spectrum, the operating point was changed and the resonance between the soliton and the mode is diminished. Accordingly, most of the spectral shift in this case results from the Raman SSFS.

### Soliton recoil and hysteresis

A change in the slope of the soliton comb line will occur when the soliton repetition frequency, *ω*_rep_, is changed (equation (1)). On account of second-order dispersion *ω*_rep_ depends linearly on the frequency offset, Ω, of the soliton spectral maximum relative to the pump frequency^19^,^40^. This frequency offset has contributions from both the Raman SSFS, Ω_Raman_, and the dispersive-wave recoil, Ω_Recoil_ (that is, Ω=Ω_Raman_+Ω_Recoil_). Accordingly, the soliton repetition rate is given by,


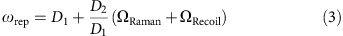


where *D*_2_ (the second-order dispersion of soliton-forming mode family at *μ*=0) is measured to be 17 kHz from Fig. 1a. Substituting for the repetition rate in the comb line expression (equation (1)) gives,


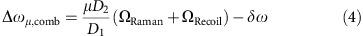


It is shown in the Methods (equation (25)) that the soliton recoil frequency has a linear dependence on the hybrid mode power,


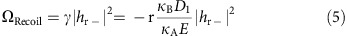


where *κ*_A_ and *κ*_B_ denote the power loss rates of the family A and family B modes, respectively; and *E* is the circulating soliton energy.

Solving equation (2) for the steady-state power in the hybrid mode at the soliton comb line frequency and using equations (4 and 5) gives the following result,


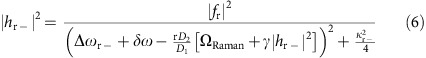


Equation (6) suggests that a bistable state and hysteresis behaviour in the dispersive-wave power is possible when varying the soliton operating point. Consistent with this possibility, it is noted that the two soliton spectra in Fig. 1b (blue and red), which show very different dispersive-wave powers, were produced at nearly identical detuning frequencies, *δω*. A more detailed survey of the dispersive-wave power behaviour is provided in Fig. 1c and is again consistent with a hysteresis behaviour versus detuning. Moreover, since the total spectral shift of the soliton is given by Ω=Ω_Raman_+Ω_Recoil_=Ω_Raman_+*γ*|*h*_r−_|^2^, a corresponding behaviour is observed in the overall soliton spectral shift (Fig. 1d). Theoretical fits are provided in Fig. 1c,d using equation (6). The fitting procedure and parameter values are provided in the Methods.

In plotting the data, the detuning frequency, *δω*/2*π*, was determined from the measured total soliton spectral shift (Ω) and pulse width (*τ*_s_) using the relation *δω*=(*D*_2_/2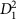)(1/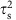
+Ω^2^). This expression is a generalization of a relationship derived elsewhere^18^. The generalization extends the shift Ω to include both the SSFS and the recoil and is derived as equation (33) in the Methods. As an aside, the pulse width is determined by fitting the soliton optical spectrum^4^.

Likewise, the recoil frequency, Ω_Recoil_, can also be extracted from the data as Ω−Ω_Raman_ by first using the soliton pulse width to determine the Raman shift using Ω_Raman_=−8*τ*_R_*D*_2_/15*κ*_A_
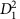

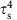
. A plot of the recoil shift determined this way versus the dispersive-wave power is given as the inset in Fig. 1c and verifies the linear dependence (equation (5)). Equation (5) is also plotted for comparison using parameters given in the Methods. As an aside, the Raman shift formula noted above is also a generalization of a result proven elsewhere^18^. Curiously, as shown in the Methods, this formula maintains its previous form in the presence of the dispersive wave.

Within narrow detuning frequency bands in the vicinity of the hysteresis both measurements and calculations show that the total cavity power (soliton and dispersive-wave contributions) can decrease with increasing cavity-pump detuning as opposed to increasing with detuning as is typical for a soliton. Under these special conditions, the pump-cavity detuning will no longer be dynamically stable on account of the thermal nonlinearity^47^. Evidence of this was observable in the current work as it was not possible to completely map out the theoretically predicted hysteresis curves.

While the present results are produced using a dispersive wave that is blue-detuned relative to the soliton spectral maximum, the hysteresis behaviour is also predicted to occur for a red-detuned dispersive wave. However, in the red-detuned case, the orientation of the curve in Fig. 1c is reversed with respect to the detuning frequency. The essential feature for appearance of the hysteresis is that the recoil advances and retreats versus detuning. As a result, the existence of hysteresis behaviour predicted in equation (6) is not limited to microresonator materials having a strong Raman SSFS. It is also predicted to occur, for example, in crystalline resonators given an appropriate avoided-mode crossing. The requirements imposed on the device and mode crossing for this to occur are discussed further below.

### Numerical simulation

**Figure 2:**
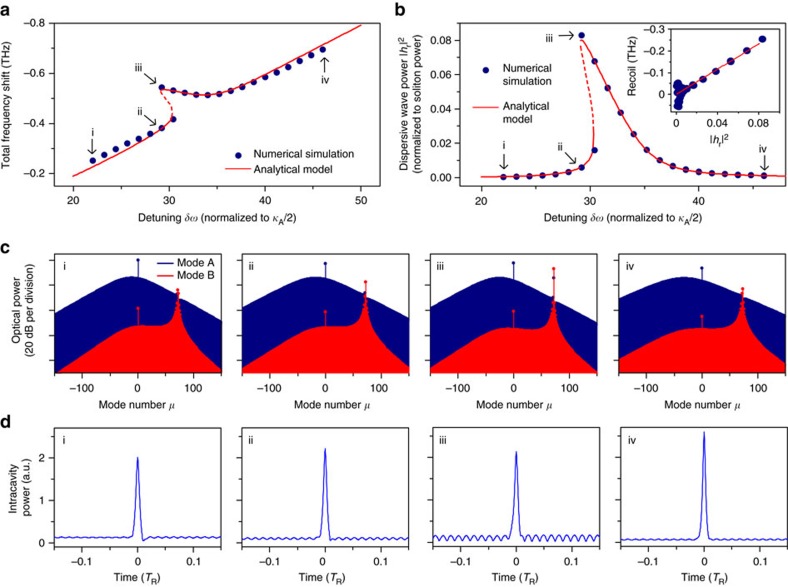
Numerical simulation and analytical model of single-mode dispersive-wave generation and recoil. (**a**) Numerical (blue dots) and analytical (red solid line) soliton total frequency shift versus cavity-pump detuning. Points i, ii, iii and iv correspond to specific soliton operating points noted in other figure panels. (**b**) Numerical (blue dots) and analytical (red solid line) dispersive-wave power (normalized to total soliton power) versus cavity-pump detuning. Inset: recoil frequency versus the dispersive-wave power. (**c**) Comb spectra contributions from the two mode families (blue: soliton forming mode family A; red: crossing mode family B). (**d**) Time domain intracavity power. *T*_*R*_ is the cavity round trip time.

To further investigate the single-mode dispersive-wave phenomena, we perform numerical simulations based on the coupled Lugiato-Lefever equations^34^,^48^,^49^,^50^,^51^ involving the soliton-forming mode family (family A) and the crossing-mode family (family B). Additional information including parameter values is provided in the Methods, but is outlined here. The two mode families are coupled using a model studied elsewhere^19^. The coupling is characterized by a rate constant *G* and is designed to induce an avoided-mode-crossing around mode index *μ*=72, similar to the experimental mode family dispersion. Figure 2 shows the results of the numerical simulation including 2,048 modes. The hysteresis behaviour in the soliton total frequency shift and the dispersive-wave power resembles the experimental observation and is also in agreement with the analytical model (Fig. 2a,b). As predicted by equation (5) (and observed in the Fig. 1c, inset), the recoil is numerically predicted to vary linearly with the dispersive-wave power (Fig. 2b, inset).

Frequency and time domain features of the soliton (blue) and dispersive wave (red) are also studied in Fig. 2c,d in units of intracavity power. They show that the dispersive-wave emerges on mode family B and consists primarily of a single mode. The single-mode dispersive wave leads to a modulated background field in the resonator with a period determined by the beating between the pump and the dispersive wave. This modulation is observable in Fig. 2d. Spectral recoil of the soliton is also observable in the numerical spectra. The combined power of mode A and B spectra in Fig. 2c is the total intracavity power.

### Soliton repetition rate quiet point

**Figure 3:**
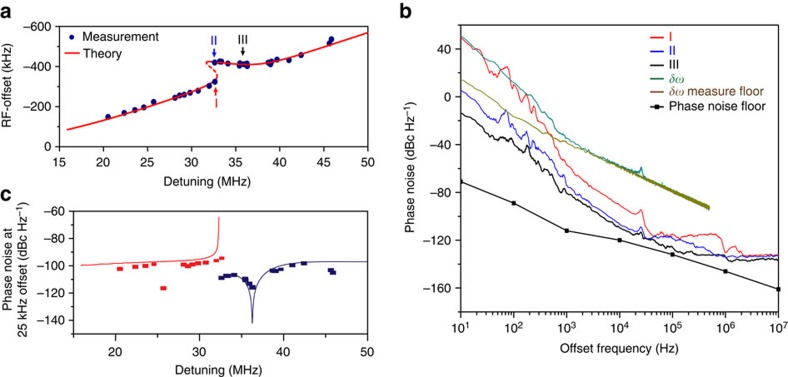
Soliton repetition frequency and phase noise measurement. (**a**) Measured (blue dots) and theoretical (red) soliton repetition frequency versus pump-cavity detuning. The offset frequency is 22.0167 GHz. The distinct soliton operating points I, II and III refer to phase noise measurments in 3b. Point III is near the quiet operation point. (**b**) Phase noise spectra of detected soliton pulse stream at three operating points shown in 3a and also the noise of the cavity-pump detuning (green) with its noise floor (brown). The black line connecting the square dots is the measurement floor of the phase noise analyser. (**c**) Phase noise of soliton repetition rates at 25 kHz offset frequency plotted versus the cavity-pump detuning. The blue and red dots (lines) denote the experimental (theoretical) phase noise of the upper (blue) and lower (red) branch operating points, respectively.

The nonlinear behaviour associated with soliton coupling to the single-mode dispersive wave can be used to suppress soliton repetition rate noise produced by coupling of pump-laser frequency noise. This noise source is suspected to be a significant contributor to repetition-rate noise in certain frequency-offset regimes^4^. From equation (3) the repetition frequency depends linearly on the total soliton spectral-centre frequency shift, Ω. However, this total shift frequency versus cavity-pump detuning has a stationary point on the upper hysteresis branch (Fig. 1d). As expected from the simple dependence in equation (3), this same stationary point is observed in measurements of the repetition frequency versus detuning (Fig. 3a). To measure the repetition frequency the soliton pulse train is directly detected and an electrical spectrum analyser is used to observe the pulse train spectrum. The theoretical prediction using analysis from the Methods is also provided for comparison.

The coupling of pump-laser frequency noise into the soliton repetition rate is expected to be minimal at the stationary point. To verify this prediction, the phase noise of the detected soliton pulse train is measured at different soliton operating points on the upper and lower branches in Fig. 3a using a phase noise analyser. Phase noise spectra corresponding to operating points I, II and III in Fig. 3a are plotted in Fig. 3b. Operating points I and II correspond to nearly identical cavity-pump detuning, but lie on different branches. As expected, operating point II in the upper branch has a lower phase noise level compared to operating point I on account of its reduced slope. Operating point III is close to the zero-slope detuning point in the upper branch. This quiet point has the lowest phase noise among the recorded phase noise spectra. At higher offset frequencies, the phase noise is shot noise limited, while at lower offset frequencies the phase noise indicates >0 dBc Hz^−1^ and is mainly contributed by frequency drift of the repetition rate.

For comparison, the phase noise associated with the detuning frequency *δω* was also measured. For this measurement, the error signal of a Pound–Drever–Hall feedback control system is operated open-loop and recorded using an oscilloscope. Its power spectral density is converted into phase noise in Fig. 3b (Supplementary Note 1). The relatively high noise floor in this measurement is caused by the oscilloscope sensitivity. Nonetheless, a noise bump at 25 kHz offset frequency originates from the laser and provides a laser-noise reference point against which comparison to the soliton phase noise is possible. The soliton phase noise at 25 kHz offset frequency noise is plotted versus detuning in Fig. 3c. The soliton phase noise is calculated in the Methods and the results are presented for comparison using the cavity-pump detuning noise level at 25 kHz offset. The dip of the phase noise occurs at the quiet point. One outlier point (red branch) is believed to have resulted from loss of lock of the phase noise analyser. For lower offset frequencies, the contributions to noise are believed to originate from thermal contributions within the resonator and are under investigation. Nonetheless, the measured noise contributions at these frequencies show a trend of reduction for operation at the quiet point.

**Figure 4:**
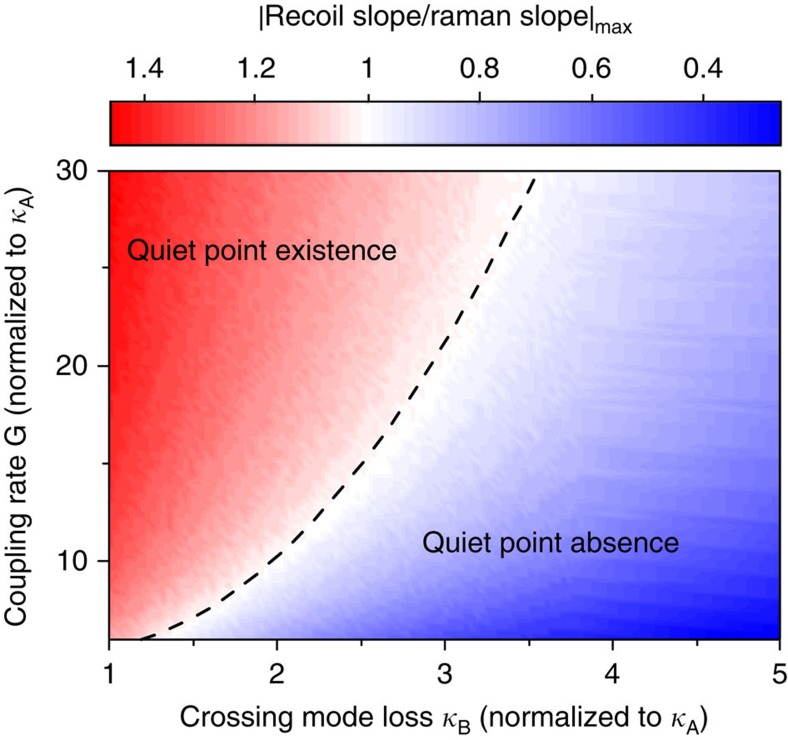
Existence study for the quiet point. The maximum ratios of |*∂*Ω_Recoil_/*∂δω*| to |*∂*Ω_Raman_/*∂δω*| at varying normalized modal-coupling rate *G* (see Methods) and normalized crossing-mode damping rate *κ*_B_ (dashed curve is unity ratio). The quiet point exists when this ratio is greater than unity (red region). Parameters correspond to a silica resonator.

An analytical study comparing the detuning response of the Raman and recoil effects was performed to determine conditions required to observe the quiet point. The quiet point occurs when the retreating soliton recoil balances the always advancing SSFS. Accordingly, Fig. 4 is a contour plot of the maximum ratio of |*∂*Ω_Recoil_/*∂δω*| to |*∂*Ω_Raman_/*∂δω*| while varying the coupling strength between the soliton-mode and crossing-mode families and the damping rate of the crossing mode (see Methods). The existence regime for observation of the quiet point corresponds to the ratio >1 shown in red. Stronger mode interaction and weaker dissipation are required to operate in this regime. Also, the impact of these parameters on the detuning range of the hysteresis is studied in the Supplementary Note 4.

## Discussion

Microfabrication control of resonator diameter, oxide thickness and wedge angle all impact the spectral placement of mode families. Numerical simulation of these families based on scanning electron micrograph measurement of resonator cross sections provides reasonably accurate dispersion maps for prediction of resonator properties. Also, process control of the resonator fabrication is sufficient to guarantee fabrication of mode families exhibiting the features shown in Fig. 1a within the 1,530–1,570 nm band.

In summary, coupling of a dissipative Kerr soliton to a single-mode dispersive wave has been shown to produce hysteresis behaviour in both the dispersive-wave power and in the soliton properties. These properties include the frequency shift of the soliton spectral centre relative to the pumping frequency and the soliton repetition frequency. The hysteresis results from the dependence of the dispersive-wave phase matching condition upon the dispersive-wave power. The hysteresis behaviour of the dispersive wave also leads to an operating point wherein coupling of laser pump frequency noise into the soliton repetition rate is greatly reduced. This reduction was modelled and measured, and the requirements for quiet point existence were also studied. The operating point for quiet soliton operation is of potential use for ultra-low-noise microwave generation.

## Methods

### Dynamical equation of hybrid mode

Equation (2) can be derived from coupled mode equations that include dispersion, mode interaction and the Kerr nonlinearity. The intracavity field of mode *μ* in the soliton-forming mode family A can be represented by 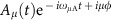, where *A*_*μ*_(*t*) is the slowly varying amplitude, *t* is the time and *φ* is the azimuthal angle along the resonator. In the rotation frame of comb frequencies *ω*_*μ*,c*omb*_=*ω*_0_−*δω*+*μω*_rep_, the intracavity field can be expressed as 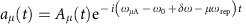. We denote the intracavity field in the crossing-mode family B as *b*_*μ*_ and express it in the same reference frame as the soliton-forming mode *a*_*μ*_. It should be noted that the relative mode number *μ* is referenced to the mode that is being optically pumped, and does not represent the actual azimuthal index. The intracavity fields can be calculated using the equations of motion with Kerr nonlinearity terms^50^,^52^ and modal-coupling terms^44^,


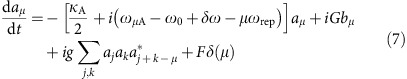



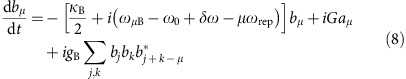


where *κ*_A,B_=*ω*_0_/*Q*_A,B_ is the dissipation rate. *g*=*ħ*
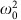
*n*_2_*D*_1_/2*πn*_0_*A*_eff_ represents the normalized Kerr nonlinear coefficient with *A*_eff_ the effective nonlinear mode area. *g*_B_ is defined similarly. *G* is the linear coupling coefficient between the two mode families^19^ and *F* is the normalized coupled laser pump field. Also, to calculate equation (2) it is not necessary to include Raman coupling terms in equations (7 and 8) since the leading-order contribution to the forcing term, *f*_r_, is from the Kerr nonlinearity.

Modal coupling causes two branches of hybrid modes to form as shown in Fig. 1a. The frequencies of the hybrid modes in the upper (+) and lower (−) branches are given by (refs 44, 53, 54),


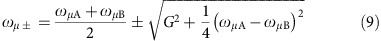


where the corresponding field amplitude of the hybrid modes is a linear combination of *a*_*μ*_ and *b*_*μ*_. In the far-detuned regime where 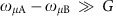, the field amplitude of the lower branch hybrid mode is approximately given by,


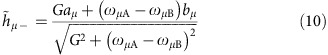


In this experiment, only one mode was observed to be near resonance with the soliton comb and that mode is assigned mode index *μ*=r. Consistent with Fig. 1a, the hybridization of mode r is assumed weak (that is, 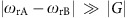 and 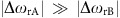) so that *b*_r_ is the dominant contribution to 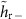. Also, since the amplitude of *b*_*μ*_ with *μ*≠r is small, the Kerr interaction summation term can be neglected in equation (8) in this calculation.

By taking the time derivative of equation (10) and then substituting using (7) and (8) the following dynamical equation results for 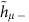,


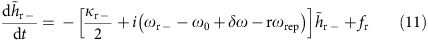


where *f*_r_ is the pumping term given by,


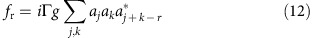


and where 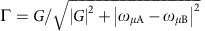 is the fraction of the family A mode in 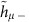 and *κ*_r−_≈*κ*_B_ is assumed for r when 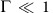. When converting equation (11) into the rotation frame of (*ω*_0_+*μD*_1_) with 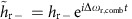, the following expression results,


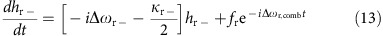


where Δ*ω*_r−_=*ω*_r−_−*ω*_*o*_−*μD*_1_ is the relative-mode-frequency of hybrid mode *h*_r−_. Equation (13) is identical to equation (2) in the main text.

### Effective pumping term

The pumping term in equation (11) can be expressed in parameters of the resonator and soliton. The soliton field envelope takes the form^3^,^18^


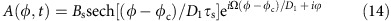


where soliton properties are: amplitude *B*_s_, angular position *φ*_c_, temporal pulse width *τ*_s_, spectral-centre frequency shift (relative to pump) Ω and phase relative to the pump laser 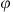. Also, this solution assumes 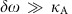. By applying the Fourier transform to *A*(*φ*, *t*), *a*_*μ*_ can be expressed in terms of the soliton properties,


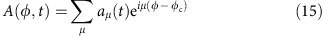



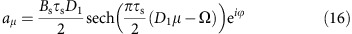


The pump *f*_r_ can therefore be derived by inserting equation (16) into equation (12). The following expression results from simplification of the summation,


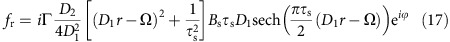


where *g* has been replaced using equation 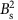

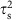
=*D*_2_/*g*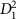, which holds for DKSs^18^,^40^ and is also verified in a section below. Finally, by using^18^ 
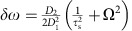
 (see derivation below), *f*_r_ can be further reduced to


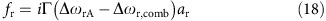


### Recoil and soliton self frequency shift

In addition to the Raman SSFS^17^,^18^, the spectral centre of the DKS is also shifted by the single-mode dispersive-wave recoil. The effect of the recoil and Raman shift can be calculated using the moment analysis method^17^,^55^. Using the Fourier transform, equation (7) is transformed into the perturbed Lugiato-Lefever equation (LLE)^50^


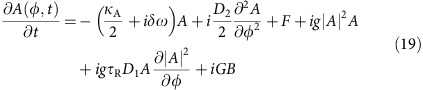


where the Raman shock term has been added^17^,^18^ and *τ*_R_ is the Raman time constant. The moment analysis method treats the soliton as a particle. The energy *E* and the spectral centre mode number *μ*_c_ are given by,


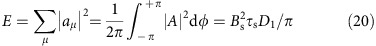



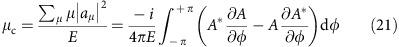


Taking the time derivative of equation (21) and substituting *∂A*/*∂t* using equation (19), the following equation of motion for *μ*_c_ is obtained,


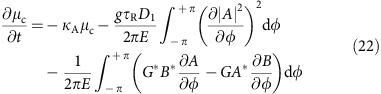


The second term on the right-hand-side corresponds to the Raman-induced frequency shift and the third term is the frequency shift caused by recoil.

The Raman term can be calculated by substituting equation (14) into the integral. When calculating the recoil term, *B* is simplified to 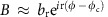 as the power in mode *B* is dominated by the near resonance mode *r*. In addition, because the integral of *φ* is over 2*π*, only 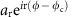 has nonzero contribution. Furthermore, equation (8) is used to relate *Ga*_r_ to *b*_r_ and finally leads to,


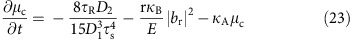


The steady-state spectral centre mode number is therefore given by,


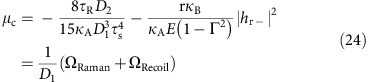


where 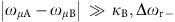 (equivalent to 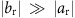) is assumed and the recoil and Raman shifts are,


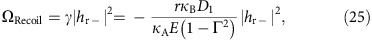



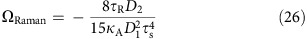


In the main text, 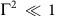 is assumed. Equation (25) is equation (5) in the main text. The form for the Raman SSFS, Ω_Raman_, is identical to the form previously derived in the absence of the dispersive-wave coupling^18^.

### Soliton parameters with Raman and mode-coupling effects

In the presence of recoil and Raman, the relations between soliton parameters in equation (14) can be derived from the Lagrangian approach^3^,^18^,^40^. In addition, the Lagrangian approach verifies the expression for Ω_Recoil_ obtained above as well as providing a path for calculation of the repetition-rate phase noise^40^. As detailed in previous literature^18^,^40^, the perturbation Lagrangian method is applied to the LLE equation of *A* (equation 19). However, now an additional perturbation term is added to account for the mode coupling to the crossing-mode family. Taking 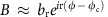, produces the following equations of motion,


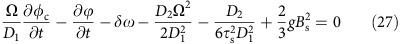



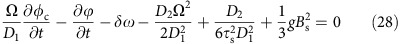



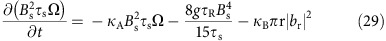



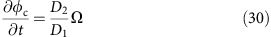



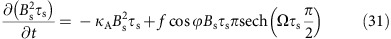


where we have assumed the mode r is far from the mode centre *μ*_c_=Ω/*D*_1_ and the coupling coefficient *G* is smaller than or around the same order of magnitude with *δω*. Also, higher order terms are neglected (Supplementary Note 2). Subtracting equation (28) from equation (27) yields


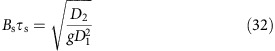


This equation was previously verified in the presence of Raman-only interactions^18^.

An additional relation between *δω*, *τ*_s_ and Ω is derived for steady state by substituting equations (30) and (32) into equation (27)


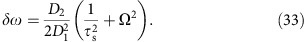


where Ω can be obtained from (29) and (32),


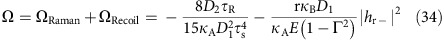


This result provides an independent confirmation of equation (24). Also, equation (33) is identical in form to an expression, which included only the Raman SSFS^18^. Significantly, however, equation (33) is more general since Ω is the total spectral centre shift provided by the combined effects of Raman SSFS and dispersive-wave recoil.

### Phase noise transfer function

The repetition rate of the soliton can be expressed as follows^19^,


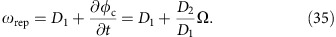


The variation in both *D*_1_ and Ω contribute to fluctuations in the repetition rate. While *D*_1_ is subject to thermorefractive noise and fluctuations from the environment, a significant contributor to fluctuations in Ω results from fluctuations in the pump-laser frequency detuning frequency, *δω*. The noise conversion from cavity-pump detuning to repetition rate can be calculated by linearizing equations (27)–(31), 57, 58, 59, 60 using the small-signal approximation^40^. Accordingly, all soliton parameters (*X*) can be expressed as *X*=*X*_0_+Δ*X*, where *X*_0_ is the steady-state value and Δ*X* is a small-signal fluctuation. For simplicity, we further denote the Raman and recoil terms in equation (29) as −8*gτ*_R_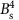/15*τ*_s_−*κ*_B_*π*r|*b*_r_|^2^≡*κ*_A_
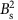
*τ*_s_*F*(*δω*) so that Ω=*F*(*δω*) is the function of detuning measured in Fig. 1d (that is, steady-state Ω versus *δω*). For simplicity, we assume this steady-state holds in the dynamical equations below. This can be shown to be true when offset frequencies (see definition below) are small compared to the cavity decay rate.

In the following derivation, *τ*_s_ in equations (27)–(31), 57, 58, 59, 60 is eliminated using equation (32). Equation (29) can therefore be expressed as


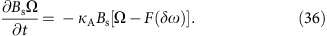


Applying the small-signal approximation and Fourier transform to equation (36) gives the result,


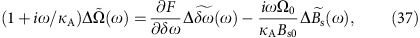


where 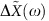 is the Fourier transform of Δ*X*, *ω* is the Fourier frequency (that is, offset frequency in the phase or frequency-noise spectrum) and where the Fourier transform of *∂*Δ*X*/*∂t* equals 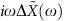. 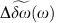 represents the cavity-pump detuning noise. Similarly, the small-signal approximation applied to equation equation (27) yields,


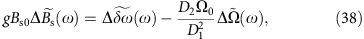


where the contribution from 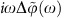 is neglected as it is of order (*ω*/*δω*) smaller compared to the leading-order terms.

In the limit of 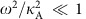 and 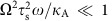, equations (37 and 38) are solved for 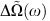 in terms of 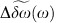. The result is substituted into the Fourier transform of equation (35) to give the following result,


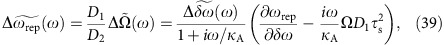


where sources of noise associated with *D*_1_ in equation (35) are ignored.

The soliton repetition rate noise can be expressed as 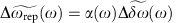 where *α*(*ω*), the noise transfer function, is the coefficient of 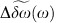 in equation (39). Accordingly, the phase noise of repetition rate is *S_φ_*(*ω*)=|*α*(*ω*)|^2^*S*_*φ*,*δω*_(*ω*).

Typically, for the resonators in this study *ω*<*κ*_A_ so that the first term in equation (39) expresses the trivial result that the slope of the plot in Fig. 3a, acts as a transfer function of fluctuations in *δω* into repetition-rate fluctutations. However, when *∂ω*_rep_/*∂δω* appoaches zero (the quiet point), the first term in equation (39) vanishes and the noise transfer function reaches a minimum determined by the second term. The phase noise plots in Fig. 3c were fitted using the same parameters as in analytical fitting in Figs 1c,d and 3a, and *∂ω*_rep_/*∂δω* extracted numerically from the fitting curves in Fig. 3a.

### Analytical model fitting and parameters

Measurements are compared with the analytical model in Figs 1c,d and 3a. Measured parameters used for the analytical model are: *κ*_A_/2*π*=2.12 MHz, *D*_1_/2*π*=22 GHz, *D*_2_/2*π*=17 kHz, *G*/2*π*=42.4 MHz. *τ*_R_=2.49 fs can be extracted from the measured Ω in the regime without the mode recoil effect (*δω*/2*π*<30 MHz and *δω*/2*π*>40 MHz). Two free parameters are used to optimize the fitting in Figs 1 and 3 and they are in reasonable agreement with the measurement: Δ*ω*_r−_=−62.2 MHz (−75±7 MHz in measurement) and *κ*_r−_/2*π*=3.6 MHz (6 MHz in measurement). The procedure for fitting is as follows: a detuning frequency, *δω*, (horizontal axis in Figs 1c,d and 3a plots) is selected. By eliminating Ω in equations (33 and 34) a single condition relating *τ*_s_ and |*h*_r−_|^2^ results. Likewise, with *δω* selected a second condition relating *τ*_s_ and |*h*_r−_|^2^ results from equation (6) by replacing Ω_Raman_ using equation (26). This pair of equations is solved for *τ*_s_ and |*h*_r−_|^2^ from which Ω is determined by equation (34) and *ω*_rep_ is determined by equation (3).

### Numerical simulations

Numerical simulations based on the coupled Lugiato-Lefever equation of mode family A and B (equation (19) and Fourier transform of equation (8)) are implemented to further validate the analytical model. The Raman term in mode family B is ignored since the power in mode family B is too small to induce Raman-related effects. Dispersion of third order and higher as well as the self-steepening effect^56^ are neglected. The simulations are implemented with the split-step Fourier method^56^ where 2,048 modes in the frequency domain are taken into account. The parameters for two mode families used in Figs 2 and 4 are *κ*_A_/2*π*=2.12 MHz, *κ*_B_/2*π*=3.4 MHz, *D*_1_/2*π*=22 GHz for mode A, *D*_1B_/2*π*=*D*_1_/2*π*+50.9 MHz for mode B, *D*_2_/2*π* =17 kHz for both mode A and B, *τ*_R_=2.489 fs, *g*=*g*_B_=9.8 × 10^−4^ rad s^−1^ and *G*/2*π*=42.4 MHz.

### Data availability

The data that support the findings of this study are available from the corresponding author upon reasonable request.

## Additional information

**How to cite this article:** Yi, X. *et al*. Single-mode dispersive waves and soliton microcomb dynamics. *Nat. Commun.* **8,** 14869 doi: 10.1038/ncomms14869 (2017).

**Publisher's note**: Springer Nature remains neutral with regard to jurisdictional claims in published maps and institutional affiliations.

## Supplementary Material

Supplementary Information Supplementary Figures, Supplementary Notes and Supplementary References
