# Supplementary material for: Single-mode dispersive waves and soliton microcomb dynamics
Source: Nat Commun. 2017 Mar 23;8:14869. doi: 10.1038/ncomms14869 (PMC5376647; doi:10.1038/ncomms14869)
Supplement: Supplementary Information — Supplementary Figures, Supplementary Notes and Supplementary References [file ncomms14869-s1.pdf]

SUPPLEMENTARY NOTE 1: EXPERIMENTAL SETUP AND DETUNING NOISE MEASUREMENT

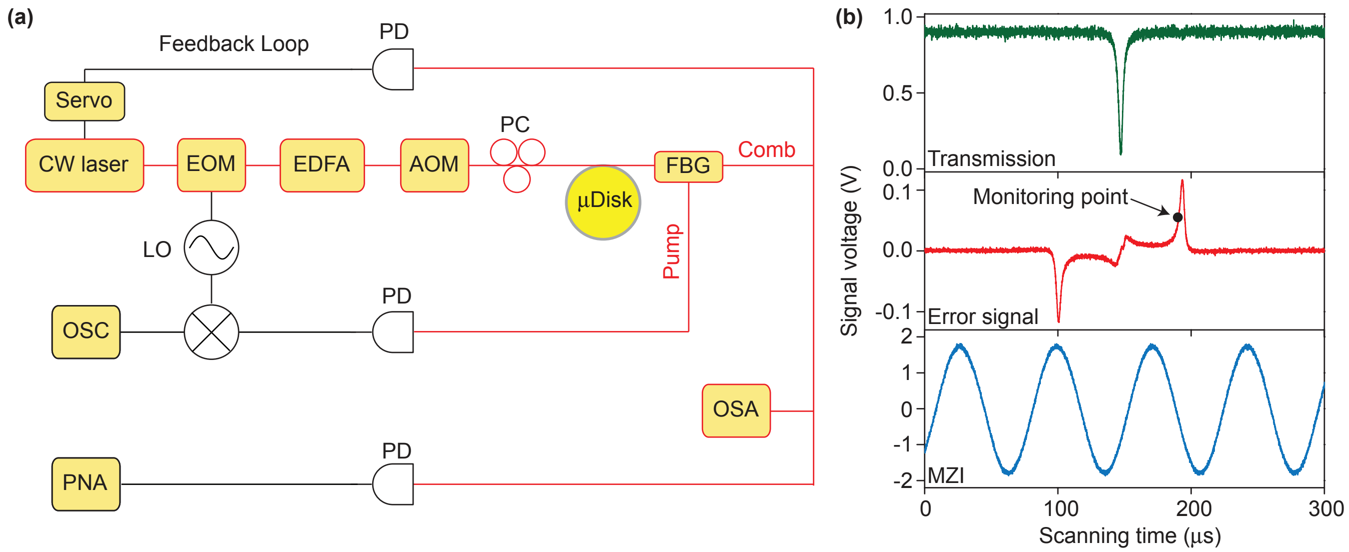

Supplementary Figure 1: **Experimental setup and details on detuning-noise measurement** (a) The experimental setup includes both the soliton generation and characterization setup[1,2] and a Pound-Drever-Hall (PDH) system operated open loop. The PDH is added to make possible the pump-cavity detuning noise measurement. Components included in the set up are an EOM: electro-optic modulator; EDFA: Erbium-doped fibre amplifier; AOM: acousto-optic modulator; PC: polarization controller; FBG: fibre Bragg grating; PD: photodetector; OSA: optical spectral analyzer; PNA: phase noise analyzer; LO: local oscillator. The OSA and and PNA are shown for completeness. They are used to measure the soliton spectrum and repetition rate phase noise. They are not involved in measuring the detuning frequency noise. (b) Measurements that illustrate the pump-cavity detuning measurement. The green trace is the measured power transmission when scanning the pump laser frequency across a cavity resonance. The pump laser is phase modulated, the transmitted signal is detected and the resulting photocurrent is then mixed with the PDH local oscillator signal to generate the PDH error signal. Upon laser scan the PDH error signal (as measured on the oscilloscope) is generated as shown in the red trace. The pump laser is filtered using the fibre Bragg grating. The monitoring point for the detuning frequency measurement is indicated by the black dot. In order to convert scanning time into laser frequency, a calibrated Mach-Zehnder interferometer (MZI) records power transmission (blue trace) on an oscilloscope. The free-spectral-range of the MZI is 40 MHz.

Solitons are generated and locked using the active-capture and locking technique[1,2]. In this method a feedback loop controls the pump laser frequency (fibre laser is used in this work) to maintain soliton power. In order to measure the detuning noise, an additional Pound-Drever-Hall (PDH) loop is embedded into the setup and operated open loop (see supplementary figure 1a). The pump frequency is red detuned relative to the cavity resonance in order to form the soliton pulse train. Moreover, the amount of cavity-laser detuning required to generate solitons is many cavity linedwidths so that the conventional PDH error signal near the resonance frequency cannot be used to monitor the detuning frequency. However, the higher-frequency PDH sideband can be tuned to reside close to the cavity resonance. Path phases in the PDH loop can be adjusted so that a PDH error signal is produced by the interaction of this sideband with the cavity resonance. When the soliton is formed, we tune the PDH local-oscillator (LO) frequency to approximately match the cavity-laser detuning. This is accomplished by monitoring the PDH error signal (see red trace in supplementary figure 1b). For this measurement the transmitted pump light is filtered from the soliton spectrum using a fibre Bragg filter. By setting this LO frequency to the indicated monitoring point, the corresponding error-signal output will convert detuning frequency to a voltage output. This output can be recorded and then analyzed to produce a noise spectrum. The calibration of voltage into frequency is performed by using the Mach-Zehnder interferometer trace (see blue trace in supplementary figure 1b). This calibration is performed on the resonator at reduced power levels where solitons do not form and where the Lorentzian lineshape of the resonator is unaffected by the Kerr nonlinearity.

SUPPLEMENTARY NOTE 2: APPROXIMATIONS IN THE EQUATIONS OF MOTION

The coupling with mode family B results in the recoil term in eq.(29) in the Methods. In eq. (27), (28), (30) and (31) (Methods), higher order terms have been neglected. Here, we list the higher order terms versus the leading order terms in each equation and therefore establish the validity conditions for these equations.

$$\frac{2\pi|G|^2\tau_\mathrm{s}D_1\Delta\omega'_\mathrm{rB}\mathrm{sech}^2(\pi\tau_\mathrm{s}(\mathrm{r}D_1-\Omega)/2)}{gB_\mathrm{s}^2(\kappa_\mathrm{B}^2+4\Delta\omega'^2_\mathrm{rB})}\leq\frac{\pi|G|^2|a_\mathrm{r}|^2}{\kappa_\mathrm{B}\delta\omega B_\mathrm{s}^2\tau_\mathrm{s}D_1}\ll 1 \tag{1}$$

$$\frac{2\pi|G|^2\tau_\mathrm{s}D_1\Delta\omega'_\mathrm{rB}\mathrm{sech}^2(\pi\tau_\mathrm{s}(\mathrm{r}D_1-\Omega)/2)[2-\Omega\tau_\mathrm{s}\pi\tanh(\Omega\tau_\mathrm{s}\pi/2)]}{gB_\mathrm{s}^2(\kappa_\mathrm{B}^2+4\Delta\omega'^2_\mathrm{rB})}\leq\frac{2\pi|G|^2|a_\mathrm{r}|^2}{\kappa_\mathrm{B}\delta\omega B_\mathrm{s}^2\tau_\mathrm{s}D_1}\ll 1 \tag{2}$$

$$\frac{|G|^2D_1\mathrm{sech}(\pi\tau_\mathrm{s}(\mathrm{r}D_1-\Omega)/2)\mathrm{F}(\Omega\tau_\mathrm{s})}{2\kappa_\mathrm{B}gB_\mathrm{s}^2\Omega}\approx\frac{|G|^2|a_\mathrm{r}|}{2\kappa_\mathrm{B}\delta\omega\Omega}\ll 1 \tag{3}$$

$$\frac{\pi|G^2|B_\mathrm{s}^2\tau_\mathrm{s}^2D_1\kappa_\mathrm{B}\mathrm{sech}^2(\pi\tau_\mathrm{s}(\mathrm{r}D_1-\Omega)/2)}{\kappa_\mathrm{A}B_\mathrm{s}^2\tau_\mathrm{s}(\kappa_\mathrm{B}^2+4\Delta\omega'^2_\mathrm{rB})}\leq\frac{4\pi|G|^2|a_\mathrm{r}|^2}{\kappa_\mathrm{A}\kappa_\mathrm{B}B_\mathrm{s}^2\tau_\mathrm{s}D_1}\ll 1 \tag{4}$$

where $\mathrm{F}(\Omega\tau_\mathrm{s})=\frac{i\pi^2}{8}[\csc^2(\frac{1+\Omega\tau_\mathrm{s}i}{4}\pi)+\mathrm{csch}^2(\frac{\Omega\tau_\mathrm{s}+i}{4}\pi)]\sim O(1)$ in supplementary equation (3) and $\Delta\omega'_\mathrm{rB}=\Delta\omega_\mathrm{rB}-\Delta\omega_\mathrm{r,comb}$. Supplementary equations (1)-(4) hold under the experimental conditions and ignoring these terms gives the equations of motion eq.(27)-(31) in the Methods.

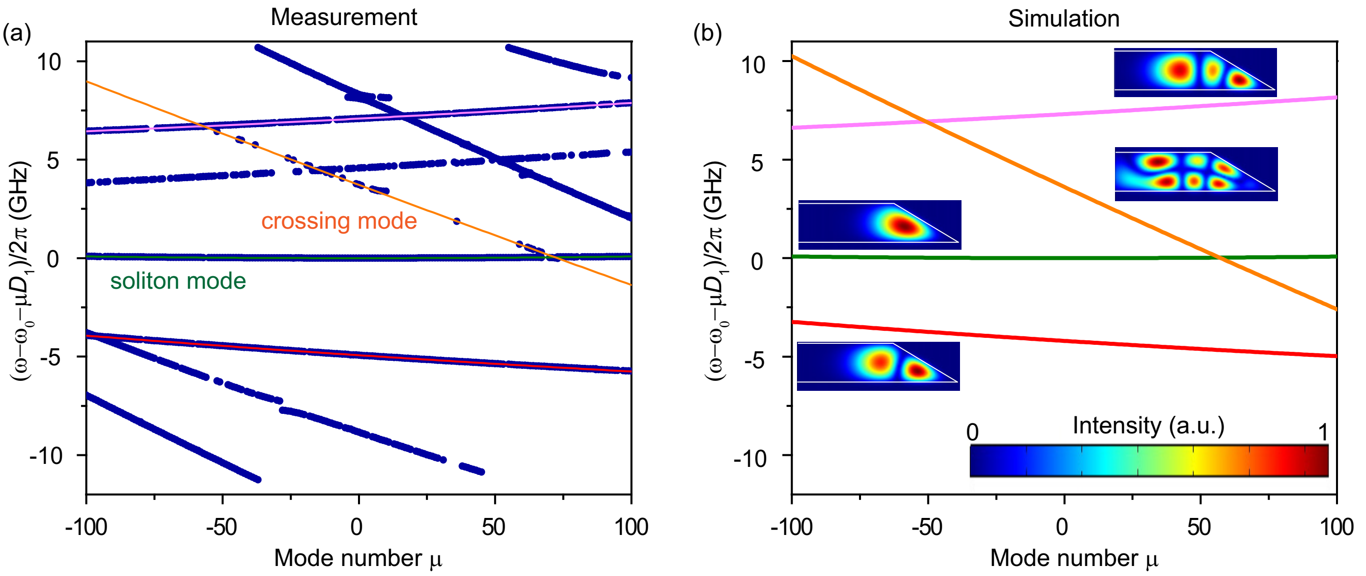

Supplementary Figure 2: **Mode spectra measurement and simulation** (a) Measured mode spectra (blue dots) and fitted lines. Green and orange lines represent fitting for soliton forming mode (mode A) and crossing mode (mode B). Red and pink lines are fitted for another two high-Q modes. $D_1/2\pi$ is 22.0167 GHz. (b) Simulated mode spectra of four transverse electric (TE) modes. Cross-sectional images of the mode spatial intensity distributions are included as insets.

SUPPLEMENTARY NOTE 3: SPATIAL MODE IDENTIFICATION

The microresonator geometry has a similar design as in our previous work[1,3], however, the actual resonator is different. To identify the spatial mode families of the soliton mode (family A) and the crossing mode (family B), a numerical simulation of the wedge resonator is used as described elsewhere[1,4]. In the simulation, the geometry of the

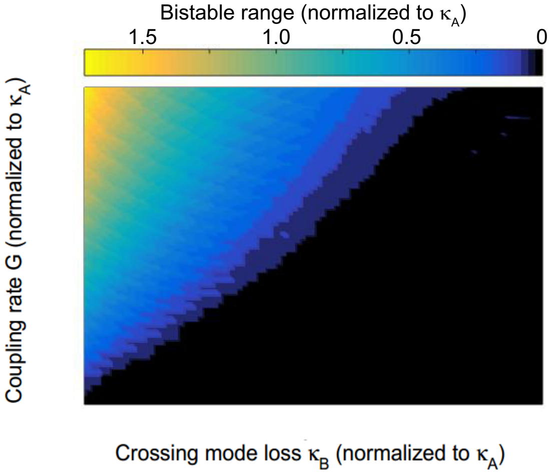

Supplementary Figure 3: **Contour plot of detuning range** Color plot of detuning range of bistability versus spatial-mode coupling strength, G, and dissipation rate of crossing mode, κ_{B}. All quantities are normalized to the dissipation rate, κ_{A}, of the soliton-forming mode.

wedge resonator was first set to the design parameters: 3 mm diameter, 30° wedge angle and 7.5 μm silica thickness. These parameters were then slightly tuned to maximize the similarity of measured and simulated mode spectra. In the simulation presented in supplementary figure 2b, the resonator has diameter 2.9826 mm, wedge angle 30.7071° and oxide thickness 7.5154 μm. The simulated mode spectrum is in reasonable agreement with the measurement. The soliton mode (family A) is identified as the fundamental transverse electric (TE) mode and the crossing mode (family B) is the TE21 mode. At the mode-crossing point in the simulation ($\mu = 57$), the absolute mode numbers for the soliton mode and the crossing mode are calculated to be 8683 and 8584, respectively.

SUPPLEMENTARY NOTE 4: DETUNING RANGE OF BISTABILITY

The detuning range of bistability is critical for observation of hysteresis behavior. Analysis shows that this range can be increased by increasing the spatial-mode coupling strength, optical quality factor or moving the mode crossing frequency closer to the pump laser frequency (smaller r). In fig. (3), the calculated detuning range of bistability is presented as a contour plot versus coupling strength, G, and the dissipation rate of crossing mode, κ_{B}. It can be seen that the range increases with G and decreases with κ_{B}. This is mainly because larger G and smaller κ_{B} enhance the dispersive wave power and therefore increase the strength of recoil and nonlinearity. In our experiment, we have measured $G/\kappa_{\mathrm{A}} = 20$ and fitted $\kappa_{\mathrm{B}}/\kappa_{\mathrm{A}} = 1.7$ and the corresponding normalized bistable range is estimated to be 0.67.

SUPPLEMENTARY REFERENCES

[1] Yi, X., Yang, Q.-F., Yang, K. Y., Suh, M.-G. & Vahala, K. Soliton frequency comb at microwave rates in a high-Q silica microresonator. *Optica* **2**, 1078–1085 (2015).
[2] Yi, X., Yang, Q.-F., Youl, K. & Vahala, K. Active capture and stabilization of temporal solitons in microresonators. *Optics Letters* **41**, 2037–2040 (2016).
[3] Yang, Q.-F., Yi, X., Yang, K. Y. & Vahala, K. Spatial-mode-interaction-induced dispersive-waves and their active tuning in microresonators. *Optica* **3**, 1132–1135 (2016).
[4] Yang, K. Y. *et al.* Broadband dispersion-engineered microresonator on a chip. *Nature Photonics* **10**, 316–320 (2016).
